# Changes in Cognitive Functions in the Elderly Living in Temporary Housing after the Great East Japan Earthquake

**DOI:** 10.1371/journal.pone.0147025

**Published:** 2016-01-13

**Authors:** Aiko Ishiki, Shoji Okinaga, Naoki Tomita, Reiko Kawahara, Ichiro Tsuji, Ryoichi Nagatomi, Yasuyuki Taki, Takashi Takahashi, Masafumi Kuzuya, Shigeto Morimoto, Katsuya Iijima, Takeyoshi Koseki, Hiroyuki Arai, Katsutoshi Furukawa

**Affiliations:** 1 Department of Geriatrics and Gerontology, Institute of Development, Aging and Cancer, Tohoku University, Sendai, Japan; 2 Department of Gerontological Nursing, Tohoku University School of Medicine, Sendai, Japan; 3 Department of Public Health and Forensic Medicine, Tohoku University Graduate School of Medicine, Sendai, Japan; 4 Division of Biomedical Engineering for Health and Welfare, Tohoku University Graduate School of Biomedical Engineering, Sendai, Japan; 5 Division of Developmental Cognitive Neuroscience, Institute of Development, Aging and Cancer, Tohoku University, Sendai, Japan; 6 Department of Infection Control and Immunology and Graduate School of Infection Control Sciences, Kitasato Institute for Life Sciences, Kitasato University, Tokyo, Japan; 7 Department of Community Healthcare and Geriatrics, Nagoya University Graduate School of Medicine, Nagoya, Japan; 8 Department of Geriatric Medicine, Kanazawa Medical University, Uchinada, Japan; 9 Institute of Gerontology, University of Tokyo, Tokyo, Japan; 10 Department of Oral Health and Development Sciences, Tohoku University Graduate School of Dentistry, Sendai, Japan; University of Perugia, ITALY

## Abstract

On March 11, 2011, Japan experienced an earthquake of magnitude 9.0 and subsequent enormous tsunamis. This disaster destroyed many coastal cities and caused nearly 20,000 casualties. In the aftermath of the disaster, many tsunami survivors who lost their homes were forced to live in small temporary apartments. Although all tsunami survivors were at risk of deteriorating health, the elderly people were particularly at a great risk with regard to not only their physical health but also their mental health. In the present study, we performed a longitudinal cohort study to investigate and analyze health conditions and cognitive functions at 28, 32, and 42 months after the disaster in the elderly people who were forced to reside in temporary apartments in Kesennuma, a city severely damaged by the tsunamis. The ratio of people considered to be cognitively impaired significantly increased during the research period. On the other hand, the mean scores of the Kessler Psychological Distress Scale-6 and Athens Insomnia Scale improved based on the comparison between the data at 24 and 42 months. The multiple logistic regression analysis revealed that frequency of “out-of-home activities” and “walking duration” were independently associated with an increase in the ratio of people with cognitive impairment. We concluded that the elderly people living in temporary apartments were at a high risk of cognitive impairment and “out-of-home activities” and “walking” could possibly maintain the stability of cognitive functions.

## Introduction

On March 11, 2011, Japan experienced unexpected strong earthquakes and tsunamis, resulting in one of the worst disasters in the nation’s history [1]. After the disaster, thousands of people were forced to live in temporary apartments because they had lost their homes. The room space of these temporary apartments was so small and limited (5 m^2^/person) that the residents were unable to continue their daily activities such as farming and fishing. In addition, the people lost their local community and opportunities for communication, which they previously had. Of the disaster victims, particularly the elderly people were at a higher risk of decrease in their mental and physical health than younger adults. Our group previously reported that cognitive functions and behavioral and psychological symptoms of dementia were significantly exacerbated in patients with Alzheimer’s disease (AD) who lived in these shelters compared with patients who did not experience the earthquake and those who remained in their own homes [2,3]. Furthermore, we reported that the ratio of elderly people with cognitive impairment was higher in the residents of temporary apartments than that of those living in other areas [4]. Therefore, it was quite important to longitudinally examine and analyze the health and cognitive condition of elderly people living in temporary apartments after the disaster. In the present study, we performed a longitudinal cohort study to clarify the health status and cognitive functions in the elderly people who lived in these temporary apartments.

## Materials and Methods

### Research Subjects

The names, addresses, and dates of birth of the source population were obtained from the city office of Kesennuma after the agreement treaty between the Tohoku University and Kesennuma city. The subject inclusion criteria were as follows: (i) lived in Kesennuma on March 11, 2011 (the day of the earthquake), (ii) aged ≥65 years on March 11, 2011, and (iii) lived in temporary apartments in Kesennuma on March 1, 2013. The total number of recruited subjects was 2,149 (male/female: 882/1,367). Their mean age at the first survey was 76.4 ± 6.0 years of age. The present study was approved by the Tohoku University ethical committee and written informed consent was obtained from all participants.

### Questionnaires on health status

We employed a set of modified questionnaires, which had already been widely used in previous studies [5,6,7]. Our questionnaire comprised of 116 questions, including items of activity of daily living (ADL), the Lawton’s instrumental ADL (I-ADL), the Kessler Psychological Distress Scale-6 (K6), and Athens Insomnia Scale (AIS). The questionnaires were distributed by mail first and were recollected by the researchers of Tohoku University and Shin Joho Center Inc. door to door one to two weeks later. If the subjects had difficulty in filling the questionnaires because of reasons such as cognitive impairment, their families or caregivers filled the questionnaires.

### Cognitive examination

Touch panel computers, on which 15 questions were already installed, were employed to examine cognitive functions [4,8,9,10]. The 15 questions evaluated memory, orientation, and pattern recognition. The best possible score was 15, and scores of ≤12 were considered to be indicative of cognitive impairment [8,9].

### Statistical methods

Logistic regression analysis was used to identify factors independently associated with change in the proportion of people with cognitive impairment after adjustment for age, gender, hypertension, diabetes and dyslipidemia by univariate analysis. We also used a two-sided t test for numerical variables and the Chi-square test for categorical variables in the analyses of longitudinal change in each category. Statistical significance was set at P < 0.05. All the statistical analyses were performed using JMP Pro (version 10.0.2,11.2).

## Results

**Fig 1:**
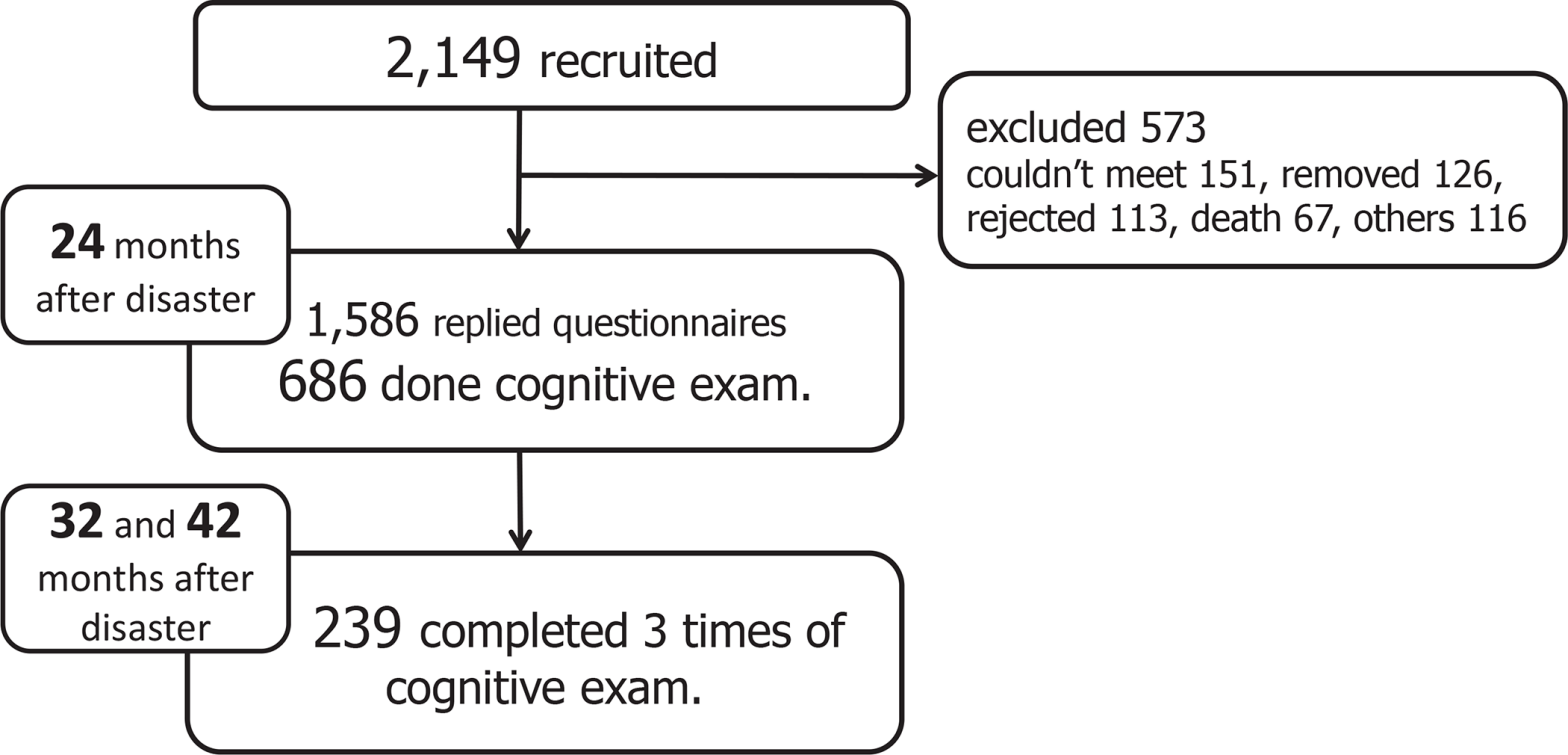
Schema of study design, sample selection, and study population.

We started this study in March 2013, when two years had passed since the disaster. One to two weeks after the questionnaires were mailed to the participants, researchers visited each temporary apartment and collected the questionnaires. The questionnaires were distributed and collected three times (24, 32, and 42 months after the disaster). Cognitive examination was also performed three times according to the same schedule. The study profile is presented in Fig 1. The number of participants at the beginning of the study was 2,139. At 42 months after the earthquake, 239 subjects had completed all the questionnaires and cognitive examinations.

**Fig 2:**
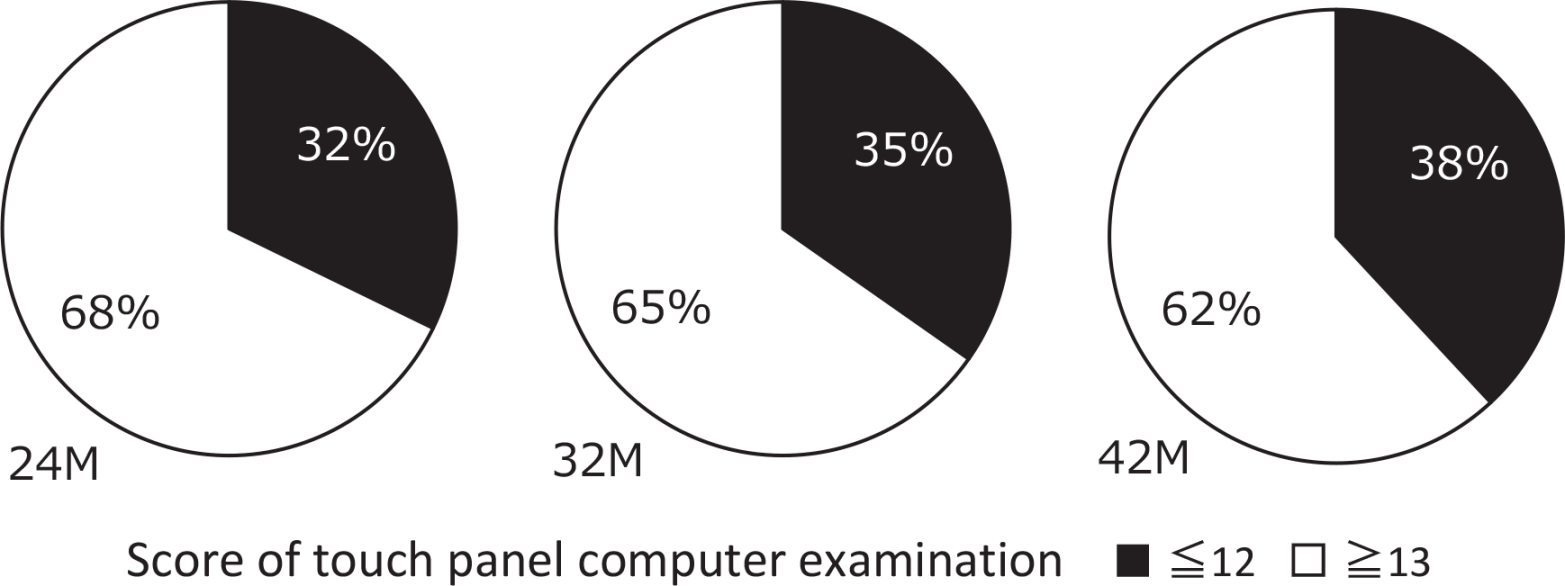
Changes in the ratio of people with normal cognitive function and those with declined cognitive function. White and black portions indicate the ratio of people with normal cognitive function (touch panel computer exam score of ≥13) and declined cognitive function (exam score of ≤12), respectively, at 24, 32, and 42 months after the disaster.

The ratio of people considered to be cognitively impaired was calculated and the change was analyzed at each time point during the study period. The ratio significantly increased during the research period by 32%, 35%, and 42% at 24, 32, and 42 months after the disaster, respectively (Fig 2). These ratios were significantly higher than those obtained in other regions, i.e., the prevalence of dementia in the population (aged ≥65 years) was reported to be 22.5% in Japan [11] and the proportion of elderly people considered to be low cognition based on a touch-panel computer’s score of ≤ 12 was reported to be 28.0% in another area [8,9,10].

Regarding psychological status, depression status as measured by K6 questions, (6.0, 5.6, and 5.6 at 24, 32, and 42 months, respectively), and insomnia condition as measured by AIS questions, (4.7, 4.6, and 4.2 at 24, 32, and 42 months, respectively) significantly improved based on the comparison between the data at 24 and 42 months (Table 1). These results suggest that the elderly people were in the process of mental recovery after the disaster.

**Table 1:** Change in each item at 24, 32, and 42 months after the disaster.

	24 months	32 months	42 months	Statistics
Ages, years	79.3±6.0	79.7±5.8	80.5±5.8	*1,*2,*3
Male (%)	102 (42.7)			
BMI (kg/m^2^)	23.4±3.0	23.4±3.1	23.6±3.6	n.s.
Grip (kg) male	24.3±8.0	24.2±7.8	24.2±7.7	n.s.
Grip (kg) female	23.7±7.7	23.5±7.7	23.4±7.5	n.s.
Lawton's I-ADL male	4.4±1.0	4.4±1.0	4.4±11.1	n.s.
Lawton's I-ADL female	6.9±1.4	7.1±1.3	7.1±1.3	n.s.
Solitude (%)	22 (11.5)	25 (13.7)	22 (11.5)	n.s.
Family bereavement (%)	99 (44.8)			
Awareness of cognitive decline after the disaster	98 (51.9)	99 (52.4)	116 (61.4)	*1,*2
Take treatment for dementia	7 (3.0)	5 (2.3)	13 (5.4)	n.s.
Score of touch-panel computer examination	12.8±2.0	12.7±2.1	12.6±2.3	n.s.
≤12 points (%)	77 (32.2)	83 (34.7)	91 (38.1)	*1,*2,*3
Athens Insomnia Scale (AIS)	4.7±3.7	4.6±3.7	4.2±3.7	*2,*3
Total score of K6 test	6.0±5.0	5.6±5.0	5.6±4.9	*1,*3
Frequency of out of home activities ≥ 3days /week (%)	133 (60.2)	135 (65.5)	141 (61.6)	n.s.
Walking duration ≥ 0.5 h/day	95 (43.8)	94 (43.3)	92 (42.4)	n.s.

*1 *P*< 0.05, Between 24 and 32 months

*2 *P*< 0.05, Between 32 and 42 months

*3 *P*< 0.05, Between 24 and 42 months

**Fig 3:**
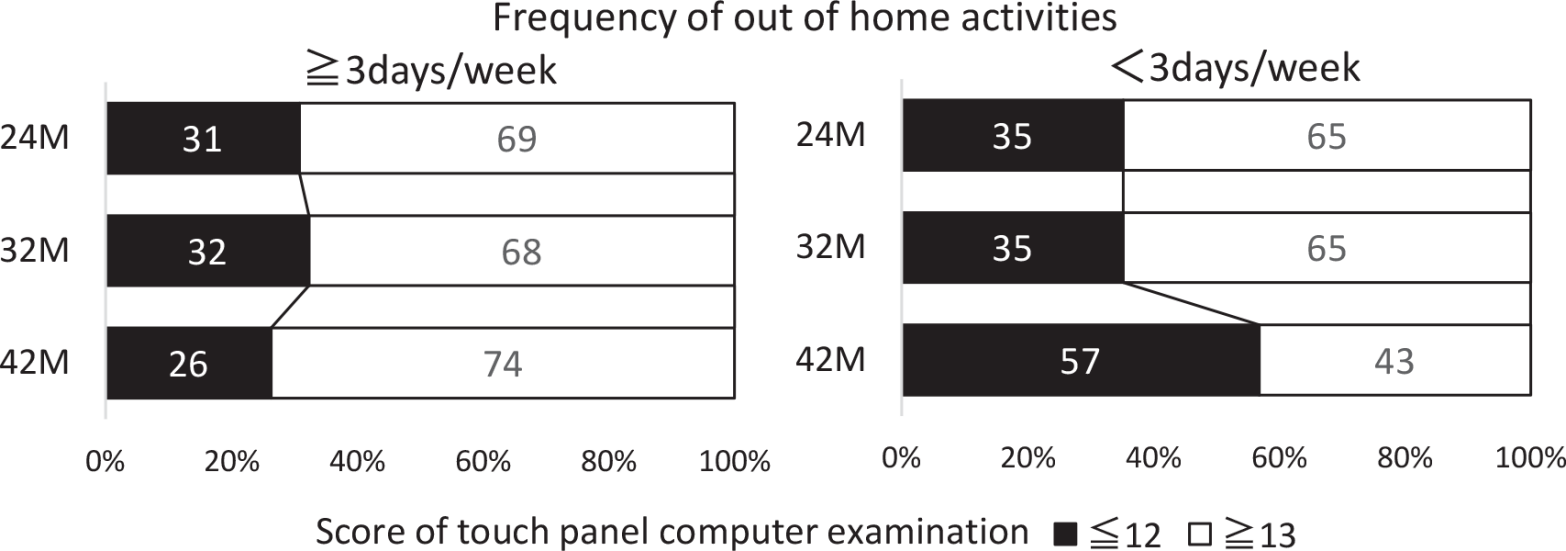
Changes in the ratio of people with normal cognitive function and those with declined cognitive function according to differences in the frequency of “out-of-home activities”. White and black columns indicate the ratio of people with normal cognitive function (touch panel computer exam score of ≥13) and declined cognitive function (exam score of ≤12), respectively. Right and left panels indicate the results of the people with the frequency of “out-of-home activities” of ≥3 day/week and <3 day/week, respectively.

In the comparison between the group in which the cognitive function declined (a touch-panel computer exam score of ≤12 at 24 months and/or that of ≤ 12 at 42 months) and the group in which the function did not decline (a touch-panel computer exam score of ≥ 13 at 24 months and/or that of ≥ 13 at 42 months), a multiple logistic regression analysis was employed to elucidate the factors associated with the change in cognition. It revealed that frequency of “out-of-home activities” and “walking duration” showed an independent association with the ratio of people with cognitive impairment (Table 2). As shown in Figs 2 and 3, the group with a lower frequency of “out-of-home activities” had a significantly increased ratio of people with impaired cognitive functions (touch-panel computer exam score ≤ 12). These results suggest that the elderly people who lived in temporary apartments had a higher risk of cognitive deterioration and opportunities for “out-of-home activities” and “walking” possibly keeps cognitive functions stable.

**Table 2:** Logistic regression analysis to identify factors independently associated to cognitive functions.

	Declined or maintained low score based on the touch-panel computer exam.	Improved or maintained high score based on the touch-panel computer exam.	Statistics
Subject number (%)	91 (38.1)	148 (61.9)	*P* = 0.0414
Age (years)	80±5.6	78.3±6.4	n.s.
BMI (kg/m^2^)	24±3.3	23.3±2.9	n.s.
Grip (kg)	23±6.5	24.6±8.5	n.s.
Athens Insomnia Scale (AIS) (at 24 months)	4.6±3.6	5.1±4.0	n.s.
Total score of K6 test (at 24 months)	6.0±4.6	6.0±5.2	n.s.
Touch-panel computer exam score (at 24 months)	12.0±2.4	13.3±1.6	*p*<0.0001
Touch-panel computer exam score (at 32 months)	12.0±2.5	13.2±1.7	*p<*0.0001
Touch-panel computer exam score (at 42 months)	10.0±2.3	13.9±0.7	*p*<0.0001
Walking duration ≥ 0.5 h/day (%) (at 24 months)	26 (31.7)	69 (51.1)	*p*<0.005
Frequency of out of home activities 3 ≥ days/week (at 24 months)	35 (41.2)	98 (72.1)	*p*<0.0001
Solitude	6 (7.0)	18 (12.9)	n.s.

## Discussion

The Great East Japan Earthquake adversely affected human lives, particularly physical and mental health, as well as access to jobs, housing, buildings, and community. Many tsunami survivors who lost their residence had to continue their lives in a limited space [1]. Not only thousands of people lost their lives due to the earthquake and tsunamis but also the mental and physical health of several survivors deteriorated during the long process of evacuation [7]. In particular, the elderly people were more susceptible to losing their life or suffering poor health after the disaster. Temporary apartments were started to be built some weeks after the earthquake. Needless to say, these apartments were much better than shelters such as gymnasiums and school halls; however, their space was so small and the communities, which the people used to have, hardly existed anymore. In these inferior conditions, there was concern regarding the physical and mental health of temporary apartment residents, particularly the elderly. Cloyd and Dyer stated that older adults are extremely vulnerable after catastrophic events through their experience with Hurricane Katrina [12,13]. Therefore, we started a survey of the health and cognition of the elderly people living in temporary apartments. Firstly, the ratio of people with low cognitive functions was significantly higher in these people than those living in the non-disaster area [4,8,9,10,11]. The ratio also increased during the research period. Our group previously reported a worsening of AD symptoms after the earthquake [2,3]. The reasons we speculate for these phenomena are as follows: (i) dramatic changes in the living space, (ii) loss of families, relatives, and friends, (iii) loss of their daily activities, and (iv) loss of communications with families and neighbors. Therefore, we are now planning to perform future studies to elucidate these issues. In addition, cerebral circulation could affect cognitive impairment because Omama et al. reported that the occurrence of cerebral infarction among elderly men was more than doubled after the disaster [14,15].

Sakuma et al. (2015) reported a high prevalence of post-traumatic stress disorder and depression in municipality and medical workers after the Great East Japan Earthquake [16]. The increase in the number of patients with seizures following the earthquake was also reported [17]. Our study indicates that K6 and AIS scores improved based on the comparison between the data at 24 and 42 months. The effects and influence of the disaster on the survivors is quite different and varied. We believe that subjects were under recovery after the disaster because the present study was conducted between 24 and 42 months after the earthquakes and tsunamis. Furthermore, the positive influence of care workers and volunteers to support the tsunami survivors in the improvement of depression and insomnia cannot be ignored.

In the multiple logistic regression analysis, frequency of “out-of-home activities” and “walking duration” were independently and inversely associated with an increase in the ratio of people with cognitive impairment. Kasper et al. (2015) reported that cognitive status in old age appears to impact on mobility and mood, rather than on involvement in out-of-home behavior connections [18]. They reported that the elderly people with AD and mild cognitive impairment (MCI) showed lower mood than cognitively healthy people. They also reported a strong positive link between mood and out-of-home behavior in patients with AD. Furthermore, the complexity of out-of-home behaviors among cognitively healthy, patients with MCI, and patients with AD was reported [19]. They concluded that cognitively demanding activities were significantly different between “MCI and cognitively healthy” and “AD and cognitively healthy” subjects. There are several studies reporting that physical activity or walking can protect against cognitive decline and dementia in the elderly people [20,21]. Karp et al. reported that a broad spectrum of activities seems to be more beneficial than to be engaged in only one type of activity to prevent dementia [22]. It is believed that “out-of-home activities” and “walking duration,” which include physical movement and communication with others, should be beneficial in the prevention or delay of dementia symptoms.

In conclusion, the cognitive functions of elderly people living in temporary apartments are at risk. To prevent dementia or keep cognition stable, we recommend involvement in “out-of-home activities” and “walking” as much as possible. As a result of our findings, we have now implemented some community programs based on “out-of-home activities” and “walking” at temporary apartments to prevent dementia and frailty.
